# Supplementary material for: Identifying Molecular Signatures of Distinct Modes of Collective Migration in Response to the Microenvironment Using Three-Dimensional Breast Cancer Models
Source: Cancers (Basel). 2021 Mar 20;13(6):1429. doi: 10.3390/cancers13061429 (PMC8004051; doi:10.3390/cancers13061429)
Supplement: Supplementary file 1 [file cancers-13-01429-s001.zip › cancers-1123664-supplementary-FINAL/Figure S1-S3 and Table S1-S2.docx]

Identifying Molecular Signatures of Distinct Modes of Collective Migration in Response to the Microenvironment using Three-dimensional Breast Cancer Models

Catalina Ardila, Vaishali Aggarwal, Manjulata Singh, Ansuman Chattopadhyay, Srilakshmi Chaparala, Shilpa Sant


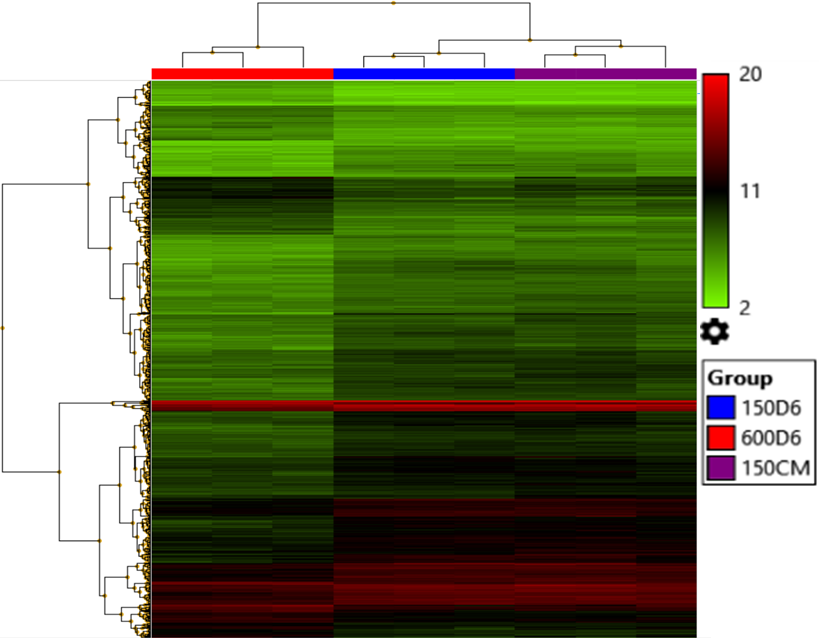


**Figure S1.** Hierarchical clustering of tumor intrinsic hypoxia-induced directional migratory microtumors (600D6), secretome induced-radial migratory microtumors (150CM), and non-hypoxic non-migratory microtumors (150D6) gene expression data. High gene expression is shown in red and low expression in green.


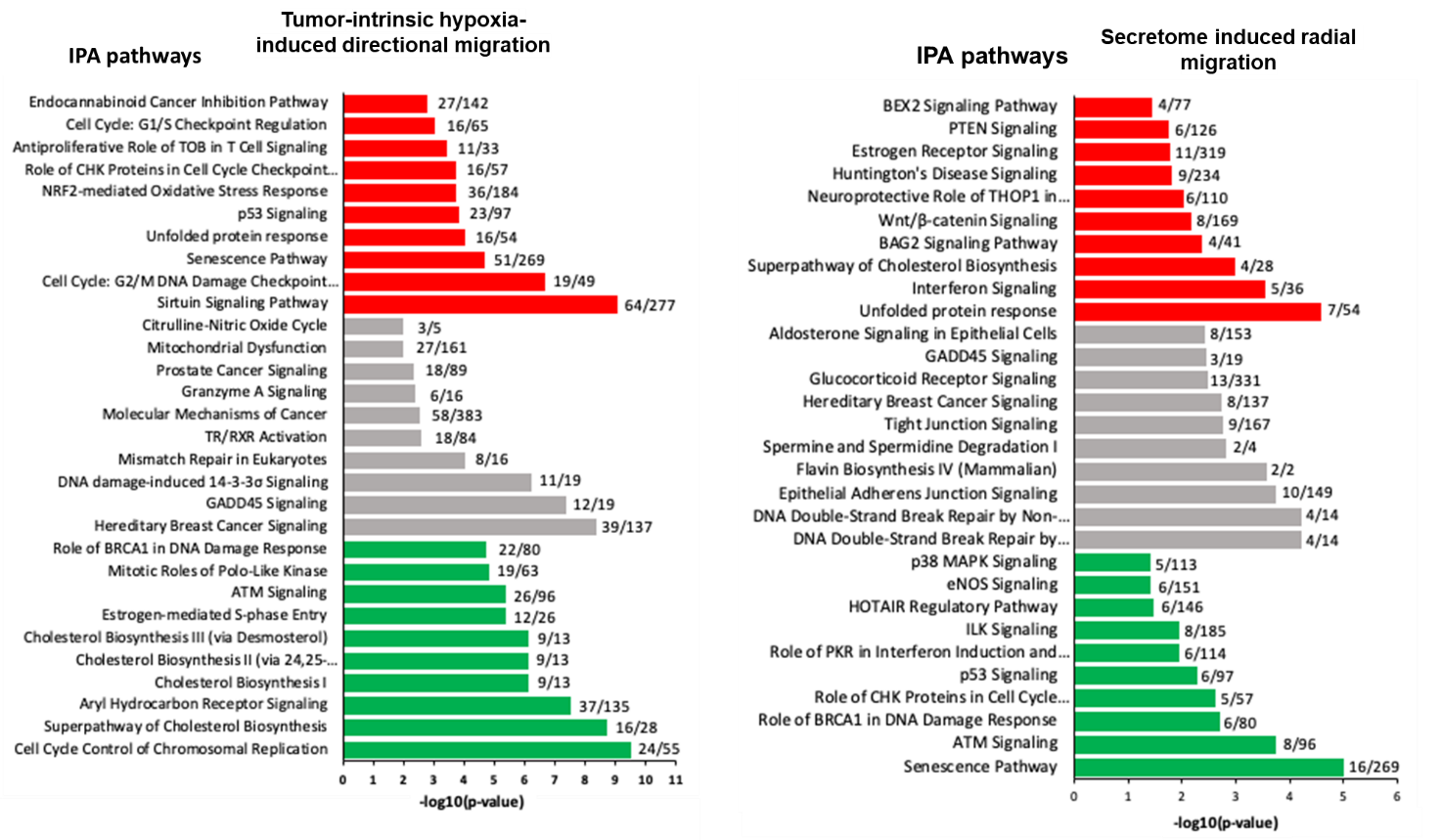


**Figure S2.** IPA pathways in Tumor-intrinsic hypoxia-induced directional migration and Secretome induced radial migration. Upregulated pathways are represented in red, downregulated pathways in green and pathways without status in IPA in grey.


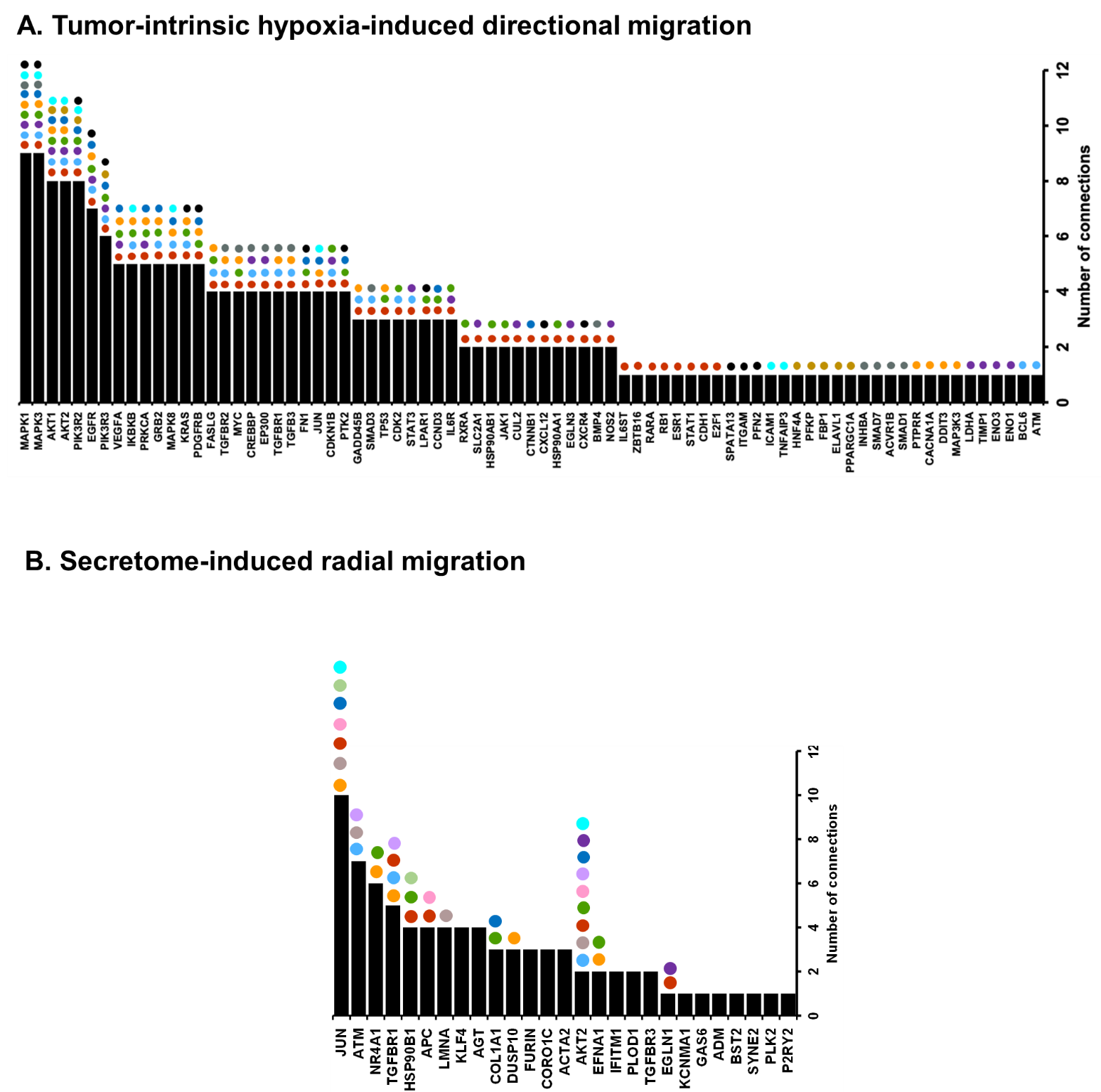


**Figure S3.** Number of connections (degree) of genes in minimum PPI network for **A**) Tumor intrinsic hypoxia induced directional migration, and **B**) secretome induced radial migration. The colored circles represent the pathways (**Fig.** **6C**, and **6F** respectively) in which each gene is participating.

**Table S1.** Seven publicly available studies used in meta-analysis.

| **GSE Accession Number** | **Study** |
| --- | --- |
| GSE3893 | Breast cancer - Invasive tumors vs Ductal carcinomas in Situ_GPL570 |
| GSE19123 | MCF7 + 4hr hypoxia (1% oxygen) vs normoxia |
| GSE29406 | MCF7 in 1% O2 10mM Lactate pH 6.7 24hr vs untreated control |
| GSE70805 | MCF7 grown 24hr in hypoxia vs normal oxygen levels |
| GSE47533 | MCF7 cultured 48hr under hypoxic conditions 1% O2 vs normoxic 21% O2 |
| GSE9649 | HMECs exposed to lactic acidosis vs exposed to hypoxia |
| GSE30019 | MCF7 exposed 24hr to hypoxia + reoxygenated 12hr vs 0hr |

**Table S2.** Top five functional terms from gene functional classification of biological processes coding for 79-gene tumor intrinsic hypoxia gene signatures, 26-gene EMT signature, 69-gene directional migration signature and 21-gene radial migration signature set using Gene Functional Classification (DAVID Bioinformatics Resources 6.8, NIAID/NIH), species–*Homo* *sapiens*, stringency–lowest.

| **Gene Signature** | **Gene Groups** | **Functional Terms** | **Fold Enrichment** |
| --- | --- | --- | --- |
| **Directional Migration** | | | |
| 79-gene tumor-intrinsic hypoxia signature | Group 1 | [Oxidation-reduction process](http://www.ebi.ac.uk/QuickGO/GTerm?id=GO:0055114) | 2.00E+01 |
|  |  | [Cellular protein modification process](http://www.ebi.ac.uk/QuickGO/GTerm?id=GO:0006464) | 3.20E+01 |
|  |  | [Peptidyl-proline hydroxylation to 4-hydroxy-L-proline](http://www.ebi.ac.uk/QuickGO/GTerm?id=GO:0018401) | 3.40E+02 |
|  |  | [Extracellular matrix organization](http://www.ebi.ac.uk/QuickGO/GTerm?id=GO:0030198) | 1.70E+01 |
|  |  | [Regulation of transcription from RNA polymerase II promoter in response to hypoxia](http://www.ebi.ac.uk/QuickGO/GTerm?id=GO:0061418) | 6.00E+01 |
|  | Group 2 | [Positive regulation of transcription from RNA polymerase II promoter](http://www.ebi.ac.uk/QuickGO/GTerm?id=GO:0045944) | 1.10E+01 |
|  |  | [Positive regulation of transcription, DNA-templated](http://www.ebi.ac.uk/QuickGO/GTerm?id=GO:0045893) | 1.60E+01 |
|  |  | [Negative regulation of fat cell differentiation](http://www.ebi.ac.uk/QuickGO/GTerm?id=GO:0045599) | 8.60E+01 |
|  |  | [Negative regulation of transcription from RNA polymerase II promoter](http://www.ebi.ac.uk/QuickGO/GTerm?id=GO:0000122) | 8.30E+00 |
|  |  | [Positive regulation of neuron apoptotic process](http://www.ebi.ac.uk/QuickGO/GTerm?id=GO:0043525) | 5.60E+01 |
|  | Group 3 | [Canonical glycolysis](http://www.ebi.ac.uk/QuickGO/GTerm?id=GO:0061621) | 2.20E+02 |
|  |  | [Glycolytic process](http://www.ebi.ac.uk/QuickGO/GTerm?id=GO:0006096) | 1.60E+02 |
|  |  | [Cell-cell adhesion](http://www.ebi.ac.uk/QuickGO/GTerm?id=GO:0098609) | 3.10E+01 |
|  |  | [Gluconeogenesis](http://www.ebi.ac.uk/QuickGO/GTerm?id=GO:0006094) | 1.30E+02 |
|  |  | [Fructose 6-phosphate metabolic process](http://www.ebi.ac.uk/QuickGO/GTerm?id=GO:0006002) | 3.50E+02 |
|  | Group 4 | [Response to hypoxia](http://www.ebi.ac.uk/QuickGO/GTerm?id=GO:0001666) | 3.70E+01 |
|  |  | [Dendrite morphogenesis](http://www.ebi.ac.uk/QuickGO/GTerm?id=GO:0048813) | 5.70E+01 |
|  |  | [Response to osmotic stress](http://www.ebi.ac.uk/QuickGO/GTerm?id=GO:0006970) | 5.80E+01 |
|  |  | [One-carbon metabolic process](http://www.ebi.ac.uk/QuickGO/GTerm?id=GO:0006730) | 3.50E+01 |
|  |  | [Glycosaminoglycan biosynthetic process](http://www.ebi.ac.uk/QuickGO/GTerm?id=GO:0006024) | 2.50E+01 |
| 26-gene EMT signature | Group 1 | [Platelet degranulation](http://www.ebi.ac.uk/QuickGO/GTerm?id=GO:0002576) | 3.30E+01 |
|  |  | [Cell activation](http://www.ebi.ac.uk/QuickGO/GTerm?id=GO:0001775) | 1.50E+02 |
|  |  | [Collagen fibril organization](http://www.ebi.ac.uk/QuickGO/GTerm?id=GO:0030199) | 4.30E+01 |
|  |  | [Response to peptide hormone](http://www.ebi.ac.uk/QuickGO/GTerm?id=GO:0043434) | 3.80E+01 |
|  |  | [COPII vesicle coating](http://www.ebi.ac.uk/QuickGO/GTerm?id=GO:0048208) | 2.80E+01 |
|  | Group 2 | [Negative regulation of cell proliferation](http://www.ebi.ac.uk/QuickGO/GTerm?id=GO:0008285) | 1.40E+01 |
|  |  | [Platelet degranulation](http://www.ebi.ac.uk/QuickGO/GTerm?id=GO:0002576) | 0.00E+00 |
|  |  | [Activated T cell proliferation](http://www.ebi.ac.uk/QuickGO/GTerm?id=GO:0050798) | 0.00E+00 |
|  |  | [Bleb assembly](http://www.ebi.ac.uk/QuickGO/GTerm?id=GO:0032060) | 0.00E+00 |
|  |  | [Oxidation-reduction process](http://www.ebi.ac.uk/QuickGO/GTerm?id=GO:0055114) | 0.00E+00 |
| 69-gene directional migration signature | Group 1 | [Cell-cell signaling](http://www.ebi.ac.uk/QuickGO/GTerm?id=GO:0007267) | 2.60E+01 |
|  |  | [Positive regulation of cell proliferation](http://www.ebi.ac.uk/QuickGO/GTerm?id=GO:0008284) | 1.40E+01 |
|  |  | [Ephrin receptor signaling pathway](http://www.ebi.ac.uk/QuickGO/GTerm?id=GO:0048013) | 3.90E+01 |
|  |  | [Platelet degranulation](http://www.ebi.ac.uk/QuickGO/GTerm?id=GO:0002576) | 3.30E+01 |
|  |  | [Axon guidance](http://www.ebi.ac.uk/QuickGO/GTerm?id=GO:0007411) | 2.10E+01 |
|  | Group 2 | [Patterning of blood vessels](http://www.ebi.ac.uk/QuickGO/GTerm?id=GO:0001569) | 8.00E+01 |
|  |  | [Positive regulation of osteoblast differentiation](http://www.ebi.ac.uk/QuickGO/GTerm?id=GO:0045669) | 3.70E+01 |
|  |  | [Viral entry into host cell](http://www.ebi.ac.uk/QuickGO/GTerm?id=GO:0046718) | 2.80E+01 |
|  |  | [Positive regulation of peptidyl-tyrosine phosphorylation](http://www.ebi.ac.uk/QuickGO/GTerm?id=GO:0050731) | 2.70E+01 |
|  |  | [Ciliary neurotrophic factor-mediated signaling pathway](http://www.ebi.ac.uk/QuickGO/GTerm?id=GO:0070120) | 2.20E+02 |
|  | Group 3 | [Peptidyl-serine phosphorylation](http://www.ebi.ac.uk/QuickGO/GTerm?id=GO:0018105) | 6.00E+01 |
|  |  | [Intracellular signal transduction](http://www.ebi.ac.uk/QuickGO/GTerm?id=GO:0035556) | 2.30E+01 |
|  |  | [Insulin receptor signaling pathway](http://www.ebi.ac.uk/QuickGO/GTerm?id=GO:0008286) | 7.20E+01 |
|  |  | [Positive regulation of glucose metabolic process](http://www.ebi.ac.uk/QuickGO/GTerm?id=GO:0010907) | 4.10E+02 |
|  |  | [Positive regulation of glucose import in response to insulin stimulus](http://www.ebi.ac.uk/QuickGO/GTerm?id=GO:2001275) | 2.90E+02 |
|  | Group 4 | [Cell division](http://www.ebi.ac.uk/QuickGO/GTerm?id=GO:0051301) | 3.20E+01 |
|  |  | [Establishment or maintenance of cell polarity](http://www.ebi.ac.uk/QuickGO/GTerm?id=GO:0007163) | 1.90E+02 |
|  |  | [Negative regulation of wound healing, spreading of epidermal cells](http://www.ebi.ac.uk/QuickGO/GTerm?id=GO:1903690) | 0.00E+00 |
|  |  | [Regulation of microtubule-based process](http://www.ebi.ac.uk/QuickGO/GTerm?id=GO:0032886) | 0.00E+00 |
|  |  | [Negative regulation of focal adhesion assembly](http://www.ebi.ac.uk/QuickGO/GTerm?id=GO:0051895) | 0.00E+00 |
|  | Group 5 | [Multicellular organism growth](http://www.ebi.ac.uk/QuickGO/GTerm?id=GO:0035264) | 0.00E+00 |
|  |  | [Cellular response to calcium ion](http://www.ebi.ac.uk/QuickGO/GTerm?id=GO:0071277) | 0.00E+00 |
|  |  | [Bleb assembly](http://www.ebi.ac.uk/QuickGO/GTerm?id=GO:0032060) | 0.00E+00 |
|  |  | [Protein dephosphorylation](http://www.ebi.ac.uk/QuickGO/GTerm?id=GO:0006470) | 0.00E+00 |
|  |  | [Regulation of kinase activity](http://www.ebi.ac.uk/QuickGO/GTerm?id=GO:0043549) | 0.00E+00 |
|  | Group 6 | [Regulation of protein tyrosine kinase activity](http://www.ebi.ac.uk/QuickGO/GTerm?id=GO:0061097) | 2.80E+03 |
|  |  | [Rap protein signal transduction](http://www.ebi.ac.uk/QuickGO/GTerm?id=GO:0032486) | 5.60E+02 |
|  |  | [Establishment of endothelial intestinal barrier](http://www.ebi.ac.uk/QuickGO/GTerm?id=GO:0090557) | 5.10E+02 |
|  |  | [Microvillus assembly](http://www.ebi.ac.uk/QuickGO/GTerm?id=GO:0030033) | 3.30E+02 |
|  |  | [Positive regulation of protein autophosphorylation](http://www.ebi.ac.uk/QuickGO/GTerm?id=GO:0031954) | 2.70E+02 |
| **Radial Migration** | | | |
| 8-gene hypoxia radial migration signature | No Enrichment | | |
| 21-gene radial migration signature | Group 1 | [Response to interferon-beta](http://www.ebi.ac.uk/QuickGO/GTerm?id=GO:0035456) | 4.70E+02 |
|  |  | [Response to interferon-alpha](http://www.ebi.ac.uk/QuickGO/GTerm?id=GO:0035455) | 4.20E+02 |
|  |  | [Response to interferon-gamma](http://www.ebi.ac.uk/QuickGO/GTerm?id=GO:0034341) | 1.70E+02 |
|  |  | [Negative regulation of viral genome replication](http://www.ebi.ac.uk/QuickGO/GTerm?id=GO:0045071) | 1.00E+02 |
|  |  | [Type I interferon signaling pathway](http://www.ebi.ac.uk/QuickGO/GTerm?id=GO:0060337) | 6.60E+01 |
